# Circulating cancer giant cells with unique characteristics frequently found in patients with myelodysplastic syndromes (MDS)

**DOI:** 10.1007/s12032-023-02064-z

**Published:** 2023-06-14

**Authors:** Abdullah Mahmood Ali, Fatima BenMohamed, Alessandra Decina, Sanjay Mukherjee, Shelley Levi, Laura Nalleli Garrido Castillo, Davide Bréchot, Joseph Jurcic, Azra Raza, Patrizia Paterlini Bréchot

**Affiliations:** 1 Division of Hematology/Oncology, Department of Medicine, Columbia University Irving Medical Center, New York, NY 10032 USA; 2 Edward P Evans MDS Center, Columbia University Irving Medical Center, New York, NY 10032 USA; 3 Rarecells Diagnostics, Faculté de Médecine Necker, 160 rue de Vaugirard, 75015 Paris, France; 4 Rarecells Inc, Alexandria LaunchLabs® at Columbia, Lasker Biomedical Research Building, 3960 Broadway, New York, NY 10032 USA; 5 University Paris Cité, 85 Boulevard Saint-Germain, 75006 Paris, France

**Keywords:** Myelodysplastic syndromes, MDS, Giant cells, Polyploid giant cancer cells, PGCC, ISET, Tumor markers

## Abstract

Myelodysplastic syndromes (MDS) are incurable diseases characterized by dysplastic hematopoietic cells, cytopenias in the blood and an inherent tendency for transformation to secondary acute myeloid leukemia (AML). Since most therapies fail to prevent rapid clonal evolution and disease resistance, new and non-invasive predictive markers are needed to monitor patients and adapt the therapeutic strategy. By using ISET, a very sensitive approach to isolate cells larger than mature leukocytes from peripheral blood samples, we looked for cellular markers in 99 patients (158 samples) with MDS and 66 healthy individuals (76 samples) used as controls. We found a total of 680 Giant Cells, defined as cells having a size of 40 microns or larger in 46 MDS patients (80 samples) and 28 Giant Cells in 11 healthy individuals (11 samples). In order to understand if we had enriched from peripheral blood atypical cells of the megakaryocyte line, we studied the Giant Cells using immunolabeling with megakaryocytes and tumor-specific markers. We report that the Giant Cells we found in the peripheral blood of MDS patients primarily express tumor markers. Our results show that Polyploid Giant Cancer Cells (PGCC), similar to those described in solid tumors, are found in the peripheral blood of patients with MDS and suggest the working hypothesis that they could play a role in hematological malignancies.

## Introduction

Myelodysplastic syndromes (MDS) are a heterogeneous group of stem cell disorders characterized by cytopenia with an inherent tendency for transformation to secondary acute myeloid leukemia (AML). MDS are incurable diseases except for < 10% individuals who can be successfully treated with allogeneic transplantation. Most therapies fail to obtain sustained responses because of rapid clonal evolution and appearance of disease resistance. Therefore, it would be important to find new predictive markers of clonal evolution and disease recurrence detectable in the peripheral blood that could be used as non-invasive approach to monitor patients and keep their disease under constant surveillance. To this aim, we applied ISET, a very sensitive approach to isolate cells larger than mature leukocytes, to the peripheral blood of patients with MDS. Through the enrichment of large cells, we found a relevant number of giant cells with one or multiple nuclei.

In solid cancers, polyploid giant cancer cells (PGCC) have been suggested to initiate tumorigenesis and tumor recurrence after therapy [1]. According to reported studies, increase in cell size may serve as a response to environmental stresses switching proliferative mitosis to intranuclear replication. This process is able to restructure the somatic genome for neoplastic transformation via formation of PGCCs. However, it is not known if formation of PGCC is restricted to the genesis of solid tumors or if it could also be involved in hematological malignancies. Previously, we documented the formation of PGCC in vitro in leukemia cells under multiple stress conditions but their occurrence in the blood of MDS patients was not demonstrated [2]. PGCC are heterogeneous cells with cell size ranging from 25 to 300 µm with one or more nuclei has been demonstrated in solid tumor biopsies and in circulation [3]. Several names have been used in the literature, referring to giant cells with one or more nuclei: polyaneuploid cancer cells (PACCs) [4], blastomere-like cancer cells [5], osteoclast-like cancer cells, circulating giant tumor-macrophages fusion or hybrid cells [6–11], and cancer-associated macrophage-like cells (CAMLs) [12] a term referred to PGCC reported to circulate in the blood of patients with solid cancers [13].

However, a high rate of cells called “dysplastic hypogranular megakaryocytes” and somehow morphologically similar to PGCC have been described in 80.3% of bone marrow samples from patients with MDS [14]. These atypical cells could circulate as rare cells and we could have found them upon concentration by the ISET treatment of peripheral blood. Actually, MDS is a clonal disorder characterized by ineffective hematopoiesis and variable degrees of dysplastic changes in the red cell, white cell and megakaryocyte series. Thus, giant cells with large or multinucleated nuclei found in patients with MDS have always been baptized indiscriminately as dysplastic megakaryocytes without systematic immune characterization.

Interestingly, the majority of the Giant Cells we have isolated from peripheral blood of MDS patients express tumor markers, raising the intriguing question of their nature and similarity with the PGCC described in solid tumors.

Although these data need further investigations, they open the way to efforts aimed at understanding the potential clinical impact of these newly described Giant Cells in patients with liquid cancers.

## Material and methods

### Patients and healthy subjects

Blood samples were obtained from patients and healthy individuals who consented to donate their sample to an institutional review board (IRB) approved tissue repository at New York Presbyterian Hospital/Columbia University Medical Center. Informed consent was obtained from all the subjects who participated in the study. This study is approved by the IRB under protocol AAAR7591 of Columbia University in accordance with the Declaration of Helsinki.

Complete blood count, percentage of peripheral blasts, serum levels of erythropoietin, ferritin, lactate dehydrogenase, and vitamin B12 were recorded for each patient. On a subset of patients, data from bone marrow measurements including the percentage of myeloblast, ring sideroblasts, and cellularity were recorded from the bone marrow aspiration performed on the same day as blood collection.

### Cell size analysis on ISET® filters

Trypsin-treated cultured cells were filtered using ISET® technology with or without blood according to the manufacturer’s instructions (Rarecells, Paris, France). Generated filters were then stained for 5 min with cytopathological staining using MERK’s Giemsa’s azur-eosin-methylene blue solution (Product code, 1,092,041,000, Sigma-Aldrich, Darmstadt, Germany).

### Isolation by size of tumor cells (ISET) filtration, cells staining and image digitalization

We have used the ISET platform (Isolation by SizE of Tumor cells, Rarecells Diagnostics, Paris, France), which is currently applied to study circulating tumor cells in patients with solid cancers, in order to eliminate by size the majority of mature blood cells. ISET concentrates cells which are larger than lymphocytes and neutrophils from a much larger volume of blood (5 to 10 mL) than the one used for peripheral blood smears.

The CE-IVD ISET® platform and its consumables were used as previously reported (Laget et al. 2017) to isolate and analyze large cells in blood. Peripheral blood samples were drawn into EDTA tubes (BD Vacutainer®, BD) with immediate gentle agitation [15]. They were then processed on the ISET® platform according to manufacturer instructions. Briefly, 5 to 10 mL of whole blood were diluted with a buffer containing 0.02% formaldehyde, incubated for 10 min at room temperature, and filtered through a filter having proprietary characteristics and 8 microns nominal pores size. The filtration pressure was optimized to − 10 kPa to preserve cell integrity. The membrane was then washed once with phosphate-buffered saline. After processing, filters were dried and stored at − 20 °C until use. Cells were stained with Giemsa for visualization of nuclei, cytoplasm and cell morphology and images were digitalized using an Olympus scanner BX-61VS. After membrane digitization, we used Olympus’ CellSens software to visualize the digital images, identify cells of interest, and measure their size. A proprietary software was developed and used for automated data processing.

### Immunocytochemistry

The filtration membrane has 10 spots, making it possible to process blood samples of 10 mL (1 mL per spot). Cells were stained with Giemsa for visualization of nuclei, cytoplasm and cell morphology and images were digitalized using an Olympus scanner BX-61VS.

Subsequently, the Giemsa stain was washed 5 min with PBS1X. Antigen retrieval was then performed for 20 min at 50 °C using EnVision™ FLEX Target Retrieval Solution High pH (50x) (Dako Omnis) (Agilent). Permeabilization was performed in Triton 0.1% PBS1X solution. The immono-cytochemistry was performed using EnVision™ G|2 Doublestain System, Rabbit/Mouse (DAB + /Permanent Red) (Agilent technologies). The spots were incubated in an ISET® ICC Staining Box, one hour at room temperature with specific antibodies diluted with DAKO Antibody Diluent (Agilent technologies). After immunostaining, cells were counterstained with hematoxylin 3 min and the spots were scanned using an Olympus scanner BX-61VS. The double digitalization allowed to visualize both cytomorphological details and immunolabeling on the same cell.

Giant Cells were arbitrarily defined as cells having a size of 40 microns (maximum diameter) or more. They were identified with Giemsa staining and counted. After immunocytochemistry, all these cells were individually analyzed by visualizing side by side the cytomorphological (Giemsa staining) and the immunostaining details.

For positive controls, we used the following cells/ cell lines: HeLa cells, MEG01, primary culture of monocyte-derived macrophages, alone or spiked in blood from healthy subjects, before filtration by ISET. A549 epithelial cell line from patient with lung carcinoma was used as a negative control for all the antibodies used. We also performed tests omitting the primary antibody, to ensure the absence of cross-reactivity in parallel with tests including the primary antibody, to guarantee the specificity of the antibody.

### Cell lines

*Megakaryoblastic cell line* (Product code, 94012401-1VL, MEG-01 HUMAN MEGAKARYOBLASTIC LEUKAEMIA) was purchased from The *European Collection of Authenticated Cell Cultures* (*ECACC*) (Sigma-Aldrich Darmstadt, Germany). MEG-01 cells were grown in standardized conditions in RPMI 1640 medium containing 10% heat-inactivated fetal bovine serum, 2 mM Glutamine, and 1% penicillin–streptomycin solution at 37 ℃ in an incubator with 5% CO_2_ atmosphere. This cell line was used as positive control for CD61 and CD41 staining.

*HeLa cell line* (HeLa; Cervical Adenocarcinoma; Human (Homo sapiens), product code ATCC-CCL-2) was purchased from American Type Culture Collection (ATTC, LGC, Molsheim Cedex, France). Hela cells were grown in standardized conditions in Dulbecco’s modified Eagle’s medium (DMEM) containing 10% heat-inactivated fetal bovine serum (FBS), and 1% penicillin–streptomycin solution at 37 ℃ in an incubator with 5% CO_2_ atmosphere. Cell culture mediums and FBS were purchased from Thermo Fisher Scientific, Les Ulis, France. Penicillin–streptomycin solution and Glutamine were purchased from Sigma-Aldrich, Darmstadt, Germany. This cell line was used as positive control for Syncytin-1, Runx2, and telomerase staining.

#### Monocytes-derived macrophages (MDM)

THP-1 cells were differentiated into macrophage-like cells by incubation in the presence of 200 ng/ml of Phorbol 12-myristate 13-acetate (PMA) for 24 h, which leads to a macrophage-like phenotype characterized by changes in morphology and increased cell surface adherence. PMA was purchased from Sigma-Aldrich, Darmstadt, Germany. This cell line was used as positive control for CD163, CD68, and CD11b staining.

### Antibodies

Antibodies used in this study are as follows: Recombinant Anti-CD163 antibody [EPR19518; abcam], recombinant Anti-CD68 antibody [EPR20545; abcam], Recombinant Anti-CD11b antibody [EP1345Y]—*C*-terminal (ab52478; abcam), CD61 (Integrin beta 3) Recombinant Rabbit Monoclonal Antibody (SJ19-09; Thermofisher scientific), CD41 mouse Monoclonal Antibody (CRC64; Thermofisher scientific), Rabbit Polyclonal Anti-Syncytin-1 antibody (Clinisciences), Anticorps mouse monoclonal RUNX2 (F-2; Santa Cruz), and Anti-Telomerase reverse transcriptase antibody (MA5-16033, Thermofisher).

## Results

### Patients

**Fig. 1:**
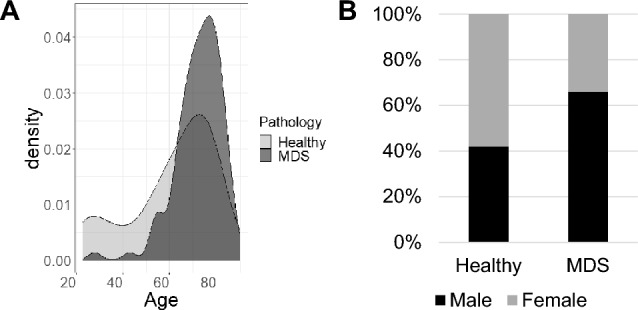
**A** Density plot showing distribution of age at samples collection between healthy subjects and MDS patients. **B** Bar graph showing percentage of males and females in healthy subjects and MDS patients

We have analyzed a total of 234 peripheral blood samples including 158 samples from 99 patients with MDS and 76 samples from 66 healthy individuals used as controls (Table 1). Longitudinal samples were available for 58 patients with MDS and 10 healthy subjects. Demographic details are provided in Table 1. Compared to controls, the patient cohort was enriched for aged and predominantly male population (Fig. 1). The cumulative amount of blood from MDS patients and healthy subjects including the longitudinal samples is 1405.5 ml and 744.7 ml respectively but the median amount of blood (10 ml) analyzed was similar in both patients and healthy subjects.

**Table 1:** Demographic data and number of subjects with giant cells of indicated size are significantly different between MDS and healthy subjects

	Cell size	Giant cells	MDS	Healthy^a^	*P*-value^b^
Number of subjects (165)			99	66	
Number of samples (234)			158	76	
Age^c^ (range)			67 (23–90)	74 (28–88)	
Sex (percent)			34F (34%); 65 M (66%)	33F (58%); 24 M (42%)	
Cell size	> = 40 um	Present	46	11	0.001
		Absent	53	55	
	> = 50 um	Present	27	5	0.002
		Absent	72	61	

^a^Demographic information not available on 10 individuals

^b^Fisher’s exact test

^c^Median age in years

### Giant cells: cell size analysis

**Fig. 2:**
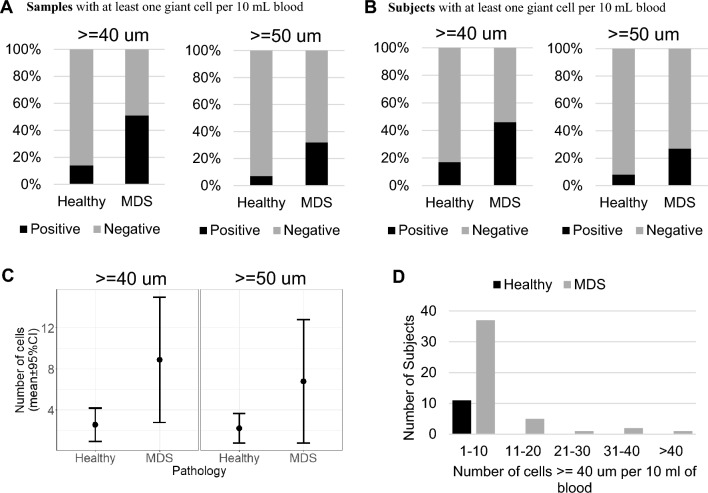
**A** Bar graph showing percentage of samples from healthy and MDS patients showing giant cells >  = 40 or >  = 50 microns. **B** Bar graph showing percentage of healthy and patients showing giant cells >  = 40 or >  = 50 microns. **C** Dotplot shows an average number of GC in healthy and MDS patients. The error bar represents 95% confidence interval. **D** Bar graph showing number of subjects showing number of GC (> = 40 um) in the indicated range

We first focused our analysis on all blood samples including longitudinal samples from MDS patients and healthy subjects. We defined Giant Cells (GC) as cells having a size (maximum diameter) equal to or larger than 40 microns. Using this definition, we identified 680 GC in 80 out of 158 samples from patients with MDS and 28 GC in 11 out of 76 samples from healthy individuals. Of the 158 samples from patients with MDS, 80 (50.6%) showed 680 cells having a size of 40 microns or larger and 50 (32%) showed 271 cells with a size equal or larger than 50 microns (Fig. 2A). Of the 76 samples from healthy subjects, 11 (14.5%) showed 28 cells having a size of 40 microns or larger and 5 (7%) showed 11 cells with a size equal or larger than 50 microns (Fig. 2A). Overall, we saw a higher percentage of MDS samples with GC compared to healthy both using a definition of GC as having a size >  = 40 and using a definition of GC as having a size >  = 50 microns (Fig. 2).

We next focused our analysis on unique patients and healthy subjects excluding repeat longitudinal samples. For this analysis we considered the first blood sample collected from patients and healthy subjects for whom we had longitudinal samples. We identified 408 GC in 46 of the 99 patients with MDS and 28 GC in 11 of the 66 healthy individuals (Fig. 2B). The 46 (46.5%) MDS patients with GC showed 408 cells having a size of 40 microns or larger. 27 of 99 MDS patients (27.3%) showed 183 cells with a size equal to or larger than 50 microns. The 11 (17%) out of 66 healthy subjects showed 28 GC cells having a size of 40 microns or larger and 5 (8%) showed 11 cells with a size equal or larger than 50 microns. Overall, we saw a significantly higher percentage of MDS patients with GC compared to healthy subjects using a definition of GC as having a size >  = 40 and using a definition of GC as having a size >  = 50 microns (Table 1 and Fig. 2B). The average number of GC of 40 microns or larger in MDS patients is 8.7 (range: 1–138) which is significantly higher than 2.5 (range 1–10) in healthy subjects (Fig. 2C). The average number of GC of 50 microns or larger in MDS patients is 6.7 (range: 1–85) which is significantly higher than 2.2 (range: 1–5) in healthy subjects (Fig. 2C).

It is important to note that in all the 11 healthy subjects in whom we identified GC, the number of GC were less than 5 per subject except for one healthy subject where we identified 10 GC (range 1–10). In the 46 MDS patients, almost a third of the patients have GC equal or greater than 10 (Fig. 2D).

### Giant cells: morphological analysis

**Fig. 3:**
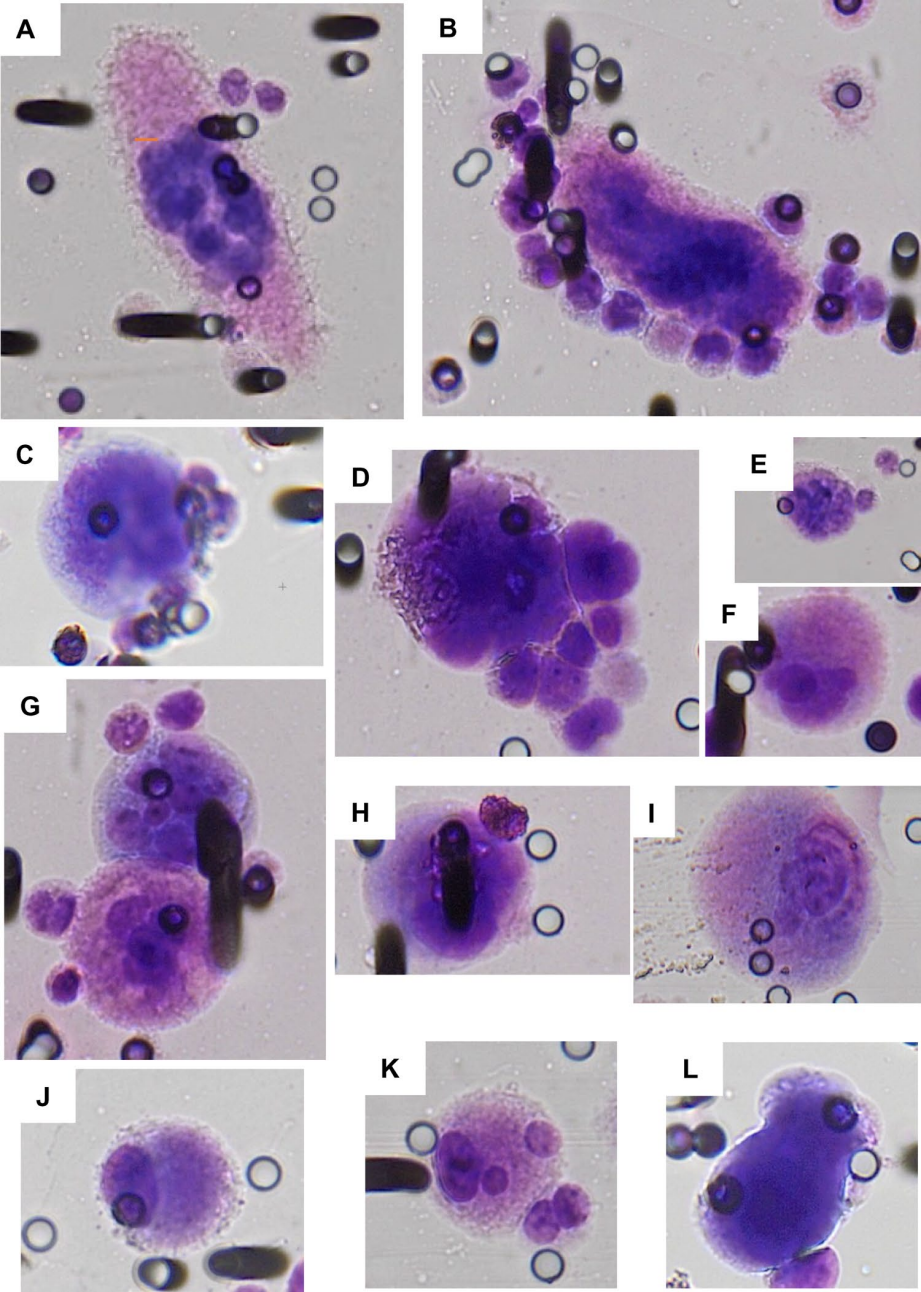
**A**–**L** A few representative pictures of giant cells from patients showing size and nuclear heterogeneity

Morphological analysis of GC was performed after Giemsa staining. Representative images of GC identified in MDS patients are shown in Fig. 3. In addition to size variability, GC displayed a clear morphological heterogeneity, including mononucleated (Fig. 3 J) and polynucleated (e.g., Fig. 3A, B, G) cells with large (Fig. 3A, F, I, J, K) or small (Fig. 3B, C, D, E, H, L) cytoplasm and cells with oval (Fig. 3A, B, L) or round (Fig. 3C, D, E, F, G, H, I, J, K) shape. The amount of nuclear material was clearly increased in all GC. For some of them it was difficult to count the number of nuclei due to the possible nuclear overlapping or presence of one or few big nuclei with heterogeneous chromatin (Fig. 3B, C, D, H, L). Interestingly, some GC were surrounded by smaller cells with blast features (Fig. 3A, B, D, G) suggesting the hypothesis that GC found in the blood of patients with MDS could give rise to blast cells. In fact, inside other GC (Fig. 3A, D, G) it was possible to identify nuclei similar to those of blast-like cells found outside the same GC.

### Giant cells: ICC immune-labeling

**Fig. 4:**
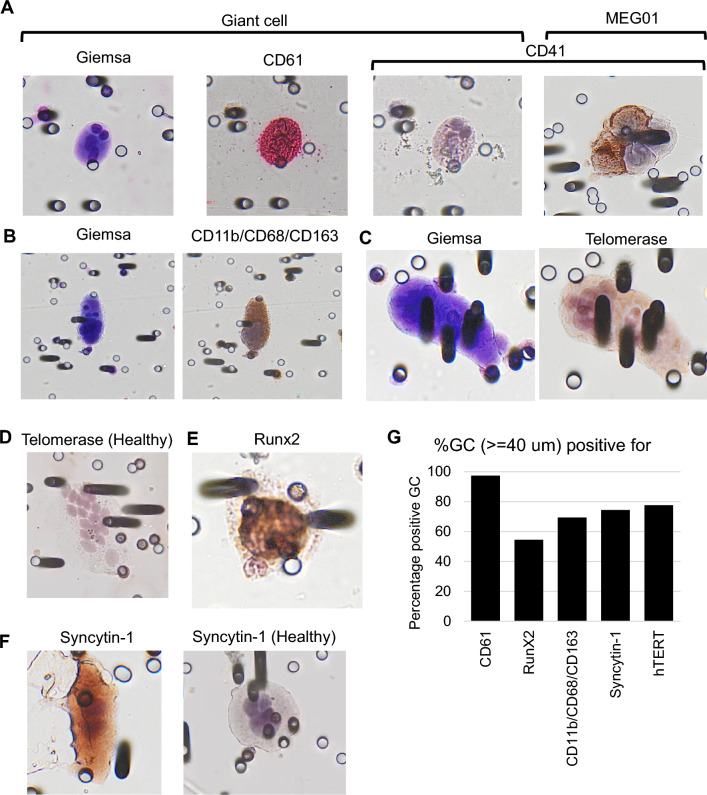
**A**–**F** Representative pictures of GC stained using Immunocytochemstry for indicated proteins from healthy and MDS patients. **A** A GC stained with Giemsa, CD61 and CD41. Only CD61 staining was positive on GC, not CD41. The positive control MEG01 cells that express CD41 showed CD41 staining. **B** A GC stained with Giemsa and a cocktail of antibody for macrophage markers CD11b, CD68, and CD163 showed positive staining. **C** A GC stained with Giemsa and Telomerase protein showed positive staining for Telomerase. **D** A giant cell obtained from a healthy subject was negative for Telomerase. **E** A GC stained positive for RunX2 transcription factor. **F** A GC from a patient was positive for Syncytin-1 protein (left) but not a GC from a healthy subject (right). **G** Bar graph showing percentage of GC positively stained for the indicated proteins

Using immunocytochemistry, we studied the expression of several proteins including CD61, CD41, Telomerase, Runx2, Syncytin-1, CD11b, CD68, and CD163 (Fig. 4). In samples from patients with MDS, CD61 marker was positive in 97.4% of GC (Fig. 4A and G). Interestingly, GC were found negative for CD41 marker (Fig. 4A), while CD61 was positive and the positive control cells MEG01 for CD41 labeling was positive (Fig. 4A), strongly suggesting that the GC we found in the peripheral blood of MDS patients are not megakaryocytes or megakaryoblasts. The macrophage panel, a cocktail of antibodies that stain CD11b, CD68 CD163, was positive in 69.4% of GC (Fig. 4B and G). The Telomerase expression was found in 77.6% of GC (Fig. 4C and G) from MDS patients but negative for GC from healthy subject (Figure D). Runx2 marker was positive in 54% of the GC (Fig. 4E and G). Syncytin-1 expression was positive in 74.4% of GC (Fig. 4F and G) from MDS patients but negative in GC from healthy subject (Fig. 4F).

In samples from healthy subjects we found GC either positive for the macrophage markers or negative for all the markers tested in the samples from MDS patients.

### Clinical correlation

We compared various clinical parameters including blast percent and complete blood counts in MDS patients with and without GC. No significant correlation between the number of giant cells and blast percentage was observed. There was no significant difference in the age of patients with and without giant cells. Also, there were no significant differences between various CBC parameters between patients with or without GC. However, it was not possible to obtain data related to treatments and/or blood transfusion before sampling, thus the correlation between the presence of GC and clinical data has to be explored in a further study.

### Longitudinal analysis of GC in MDS patients and healthy subjects

**Fig. 5:**
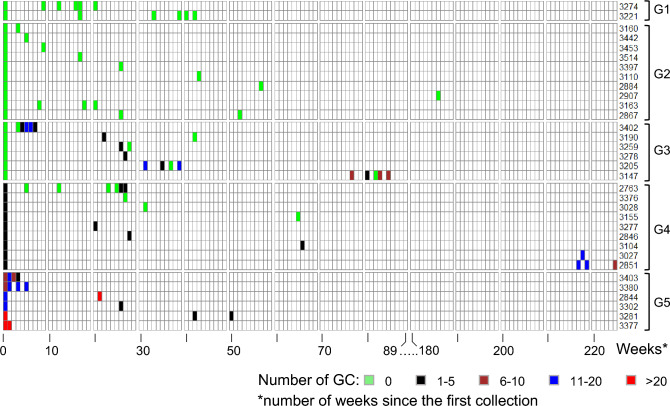
A heatmap showing number of GCs in healthy and MDS patients on whom longitudinal samples were available. Each vertical line represents one week of time, and each row represents one subject. The timeline between 90 and 179 weeks is not plotted as there are no samples in that timeline. See text (Results) for further explanations

Longitudinal samples were available for 33 subjects, two healthy and 31 MDS patients (Fig. 5). Of the 33 subjects, 19 (58%) subjects had at least 1 additional time-point and 14 (42%) had more than one time-point including 10 (30%) with greater than or equal to 4 time-points with maximum time between first and last collection up to 225 weeks (Fig. 5). Cumulatively, we have collected 10 additional samples on the two healthy subjects covering a period of 42 weeks since the first collection. No GC’s were observed in all the 12 samples in healthy subjects (G1; Fig. 5).

Within MDS patients, in 10 subjects, no GC’s were detected on initial and subsequent collections (G2; Fig. 5) but in 6 subjects, no GC’s were detected on initial collection but GC number increased on subsequent collections (G3; Fig. 5). In 9 patients, where we found at least 1 GC in the first collection, only 3 showed no GC on subsequent collection, rest had the GC number either same or increased over time (G4; Fig. 5). In 6 patients, where we identified a high number of GC on initial collection, had their GC numbers remained high in subsequent collection (G5; Fig. 5). Overall, we observed, as a trend, a consistent pattern of GC numbers in longitudinal analysis (Fig. 5). In particular, 6 of 16 MDS patients with no GC at the first sampling showed GC at subsequent samplings (Fig. 5, G2 and G3) and only one of the 6 patients had more than 5 GC. Furthermore, only 2 MDS patients with GC at the first sampling showed no GC at the following ones (Fig. 5, G4 and G5). Importantly, all MDS patients with 6 or more GC at the first sampling showed GC at the following ones (Fig. 5, G5).

## Discussion

In this study, we have found circulating Giant Cells with unique characteristics in a high proportion (46%) of patients with MDS. These cells were detected by eliminating the vast majority of mature blood cells using ISET to concentrate the larger cells and analyze them. Although the cytomorphological aspects of some of these GC are similar to those of atypical megakaryocytes and megakaryoblasts, the GC we describe express tumor markers: telomerase, syncytin and Runx2, and macrophage markers which are not expressed by cells of the megakaryocyte series. Thus, given their tumor-like characteristics, these new GC found in the peripheral blood of MDS patients should be further investigated to explore the fascinating hypothesis that they could represent in liquid tumors counterpart of PGCC described in solid cancers.

We thought that the giant cells we found could be morphologically assessed as atypical megakaryocytes. In the literature, the observation of megakaryocytes in peripheral blood is extremely rare. Ku et al. reported a single megakaryocyte in a patient with Myelofibrosis (MF) and leukoerythroblastosis [16]. The image is similar to cells we also detected (data not shown), however the Authors did not perform any labeling to confirm the megakaryocyte phenotype. Nakai et al. described another single megakaryocyte in the peripheral blood of a patients with Myelofibrosis, essential thrombocythemia and leukoerythroblastosis [17]. Garg et al. described 4 cases with macrocytic or microcytic anemia, each with one megakaryocyte in the peripheral blood [18]. Guiying Li et al. described small-sized megakaryocytes in the circulation of a patient with pseudo-hyperkalemia following splenectomy [19]. Thus, reports of circulating megakaryocytes are rare and their number extremely low, however we need to consider that we have concentrated the cells larger than mature leukocytes from larger amounts of blood (5 to 10 ml) than what is analyzed in hematological smears.

In order to understand if the GC we found are atypical megakaryocytes or not, we have used macrophage and tumor-specific markers and have applied a specific approach allowing to examine, side by side, the cell morphology and its immunolabeling by ICC (immunocytochemistry). In fact, immunofluorescence does not allow to explore in detail the morphological characteristics nor ICC which, used alone, covers the cytomorphological details.

The vast majority of the Giant Cells that we have identified were found positive for the CD61 marker. CD61, or integrin beta 3, also known as GPIIIa, is a glycoprotein of 105 kD found on platelets, monocytes, endothelial cells, smooth muscle cells, B cells, macrophages, mast cells and fibroblasts. Only the coexpression of CD61 with CD41 is considered specific of megakaryocytes. However, the GC we tested for both markers and which were positive for CD61, were negative for CD41, making it impossible to confirm their megakaryocyte origin.

Nearly 70% of GC with a size of 40 microns or larger were positive to a cocktail of macrophage markers (CD11b, CD68 CD163). This is suprising as megakaryocytes are not known to express macrophage markers and we did not find any report on the expression of macrophage markers in megakaryocytes. Furthermore, Syncytin expression was found in approximately 70% of the GC detected in MDS patients. Syncytin-1 is a cell–cell fusion protein whose function is best characterized in placental development [20, 21]. Syncytins are considered as “new” genes in mammalian species and these genes are derived from endogenous retroviral elements and obtained novel functions through a process of convergent evolution [20, 21]. This finding is intriguing as it has been described that PGCC often derived from fusion of a macrophage cell with an epithelial tumor cell. The expression of syncytins in megakaryocytes is not known and has never been described. The expression of syncytin and macrophage markers in the majority of GC we found in MDS patients let us to conclude that they are not megakaryocytes.

We also found that RunX2 is expressed in 54% of the GC. The *RUNX2* gene encodes a transcription factor that acts as a “master switch,” regulating genes involved in the development of several tissues including bones, teeth, and cartilage. However, it has also been demonstrated that RUNX2 is involved in tumor cells invasion, especially in bone metastases and in the development of malignant tumors. Thus, RUNX2 expression in the GC we found in the blood of MDS patients could be linked to the tumor characteristics of the GC, increasing the probability for the GC to be, in fact, PGCC.

Finally, 77.6% of the GC strongly expressed telomerase. Telomeres maintain genomic integrity in normal cells, and their progressive shortening during successive cell divisions induces chromosomal instability. In the large majority of cancer cells, telomere length is maintained by telomerase. Thus, telomere length and telomerase activity are crucial for cancer initiation and the survival of tumors [22]. In fact, telomerase hyperexpression is found in tumor cells or stem cells. Telomerase activity has been reported to be dysregulated in myelodysplastic syndrome (MDS) [23]. Furthermore, a report demonstrated the presence and abundance of extremely short telomeres in MDS. Critically short telomeres length is associated with bone marrow blasts in MDS and is an independent prognostic factor for PFS and OS [24]. In this context, it is difficult to understand the function of telomerase hyperexpression in GC. They seem to be non-proliferating cells, however their content of multiple nuclei and images (see Fig. 3D) resembling the budding tumor cells from PGCC described in [2] could be related to a pathological telomerase expression. In any case, telomerase hyperexpression has never been described in megakaryocytes, with the exception of apoptotic cells in the bone marrow of patients with Myelofibrosis [25].

Longitudinal analysis of samples from healthy patients indicated a consistent pattern of GC. In general, patients or healthy subjects who did not have any GC in initial collection did not show them in subsequent collection when followed for up to 200 weeks. Patients who showed GC on initial collection consistently showed GC on subsequent collections, with some patients showing an increase in GC overtime. The pattern we observed in our study of repetitive samples is consistent with observations in the field of circulating rare cells. In fact, even with a highly sensitive approach, the probability to find rare cells depends on their frequency in blood. The lower limit of detection (LLOD) of ISET is one tumor cell in 10 ml of blood [15]. If the number of rare cells falls below 500 in the average 5000 ml blood of an adult subject, the assay can be negative or intermittently positive. On the other hand, if the number of GC is higher than 500 in the whole blood volume, then the probability to find them in subsequent sampling is higher. However, these considerations have to take into account the variability of the number of GC possibly related to the disease’s progression, and to treatment, including transfusions. In our study, as mentioned above, we could not perform an analysis to relate these observations to outcomes such as survival and progression to leukemia.

In conclusion, we have described novel circulating giant cells with unique characteristics in patients with MDS. These GC are not megakaryocytes and express tumor markers and macrophage markers, thus suggesting the stimulating hypothesis that they could be analogous to the PGCC found in patients with solid cancers and thought to be at the origin of tumor formation and tumor recurrence. Further in vitro studies are needed to explore their proliferative capacity, their resistance to treatment and their possible ability to generate blasts cells by budding. Animal studies could evaluate their tumorigenic/leukemic potential. In patients, their presence and phenotype in the bone marrow should be assessed, as well as the correlation of their presence in peripheral blood with clinical data and disease evolution.

Ultimately, our findings provide more questions than answers but open a new field of investigations aimed at understanding if the GC we detected in MDS patients can be considered the equivalent of PGCC described in solid cancers.

## Data Availability

The datasets generated during and/or analyzed during the current study are available from the corresponding authors on reasonable request.

## Code availability

The code described to identify cell size and shape is proprietary.
